# Beyond Adherence Thresholds: A Simulation Study of the Optimal Classification of Longitudinal Adherence Trajectories From Medication Refill Histories

**DOI:** 10.3389/fphar.2019.00383

**Published:** 2019-04-26

**Authors:** Samuel S. Allemann, Dan Dediu, Alexandra Lelia Dima

**Affiliations:** ^1^Health Services and Performance Research (HESPER EA 7425), University Claude Bernard Lyon 1, Lyon, France; ^2^Pharmaceutical Care Research Group, University of Basel, Basel, Switzerland; ^3^Collegium de Lyon, Institut d’Études Avancées, Lyon, France; ^4^Laboratoire Dynamique Du Langage UMR 5596, Université Lumière Lyon 2, Lyon, France

**Keywords:** medication adherence, compliance, sliding window, computer modelling, methods, cluster analysis, non-parametric

## Abstract

**Background:** The description of adherence based on medication refill histories relies on the estimation of continuous medication availability (CMA) during an observation period. Thresholds to distinguish adherence from non-adherence typically refer to an aggregated value across the entire observation period, disregarding differences in adherence over time. Sliding windows to divide the observation period into smaller portions, estimating adherence for these increments, and classify individuals with similar trajectories into clusters can retain this temporal information. Optimal methods to estimate adherence trajectories to identify underlying patterns have not yet been established. This simulation study aimed to provide guidance for future studies by analyzing the effect of different longitudinal adherence estimates, sliding window parameters, and sample characteristics on the performance of a longitudinal clustering algorithm.

**Methods:** We generated samples of 250–25,000 individuals with one of six longitudinal refill patterns over a 2-year period. We used two longitudinal CMA estimates (LCMA1 and LCMA2) and their dichotomized variants (with a threshold of 80%) to create adherence trajectories. LCMA1 assumes full adherence until the supply ends while LCMA2 assumes constant adherence between refills. We assessed scenarios with different LCMA estimates and sliding window parameters for 350 independent samples. Individual trajectories were clustered with kml, an implementation of k-means for longitudinal data in R. We compared performance between the four LCMA estimates using the adjusted Rand Index (cARI).

**Results:** Cluster analysis with LCMA2 outperformed other estimates in overall performance, correct identification of groups, and classification accuracy, irrespective of sliding window parameters. Pairwise comparison between LCMA estimates showed a relative cARI-advantage of 0.12–0.22 (*p* < 0.001) for LCMA2. Sample size did not affect overall performance.

**Conclusion:** The choice of LCMA estimate and sliding window parameters has a major impact on the performance of a clustering algorithm to identify distinct longitudinal adherence trajectories. We recommend (a) to assume constant adherence between refills, (b) to avoid dichotomization based on a threshold, and (c) to explore optimal sliding windows parameters in simulation studies or selecting shorter non-overlapping windows for the identification of different adherence patterns from medication refill data.

## Introduction

Medication adherence is frequently estimated based on electronic healthcare data (EHD), such as prescription, dispensing, and claims databases. Numerous variations of the “medication possession ratio” (MPR) or “proportion of days covered” (PDC) are commonly reported as aggregate or “point” estimates of medication availability for a person over a given observation period (Dima and Dediu, 2017). Moreover, these estimates are often dichotomized at a threshold to discriminate “adherence” from “non-adherence.” A threshold of 80% has been proposed for a range of diseases, such as Schizophrenia, Diabetes, Hypertension, Hyperlipidemia and Chronic Heart Failure (Karve et al., 2009). In these studies, adherence thresholds over long time periods show only a modest prediction accuracy for clinical outcomes (Hansen et al., 2009).

The low prediction accuracy may reflect loss of information regarding the process of adherence, a process which may vary substantially across time (Steiner, 2016), and prototypically includes three phases: initiation, implementation, and non-persistence (Vrijens et al., 2012). Low adherence, when calculated across all three phases, could reflect delayed initiation of a treatment, incorrect implementation, or premature discontinuation (Vrijens et al., 2012). Patients may also have variable adherence during the implementation phase, and some temporal sequences of deviations from the prescribed regimen may be more detrimental to treatment effectiveness and safety compared to others. Characterizing patients based on an overall adherence estimate across this phase and its threshold-based dichotomization does not capture these temporal variations and therefore, may not reflect appropriately the impact of adherence on clinical outcomes. In contrast, characterizing patients based on temporal adherence trajectories is useful in many clinical and research contexts. In a clinical setting, it can guide decision-making regarding medical treatment, or behavioral support for medication use. In research, trajectories can be used for exploratory analysis of adherence patterns, as implicit predictor or covariant of outcomes, or as outcome itself (Bijlsma et al., 2016).

Several methods have shown promise in describing adherence longitudinally and classifying patients based on EHD. Short-term estimates of medication availability predicted outcome measures on corresponding time intervals better than estimates over a longer time period (Bryson et al., 2007; Nichols et al., 2015). Calculating multiple estimates of medication availability over shorter periods captured within-patient variation over a longer treatment duration (Bijlsma et al., 2016; Souverein et al., 2017). This approach is commonly described as “moving average” or “sliding windows,” and has been used in numerous fields, such as economics, finances, genomics, and electronics. It is particularly appropriate for estimating trajectories from data that have not been sampled at the same fixed time points for all subjects, which is the case for EHD. For such data, a summary measure is calculated for a specific observation period (window) based on the raw data available within that window, in order to reduce measurement error due to variations in sampling moments. Windows typically have the same duration (window length), and move (slide) forward at a constant rate (lag time or step length), which results in varying degrees of overlap between windows. Trajectory-based models have gained traction in psychology, medicine, and criminology (Nagin and Odgers, 2010) and have recently been proposed as a method to classify patients based on their longitudinal adherence trajectories (Franklin et al., 2013). These models empirically identify clusters of individuals following similar trajectories and the resulting groups can then be used as predictors or dependent variables (Genolini et al., 2015), for example to examine causes and consequences of (non-)adherence.

The challenge with this approach is to identify clusters that capture meaningful differences between individuals in terms of their temporal adherence patterns, and classify individuals accurately based on the available data. With real-world data, neither the “real” clusters nor the allocation of individuals to those clusters are known. Simulation studies offer the possibility to assess the performance of a variety of methods and parameters in relation to a known state (Burton et al., 2006). In adherence research, simulation studies have been used to estimate pharmacokinetic properties (Ding et al., 2012; Pellock and Brittain, 2016) or the impact of interventions to improve adherence (Slejko et al., 2014; Volino et al., 2014; Piette et al., 2015). Optimal methods of summarizing longitudinal adherence and ideal parametrization of sliding windows to identify underlying patterns have not yet been established for this type of data. Methods have been suggested to select optimal window size and overlaps in various fields (Chu, 1995; Pesaran and Timmermann, 2007; Rossi and Inoue, 2012; Gusnanto et al., 2014). To ensure best use of these promising classification methods, it is necessary to test how well they are able to identify known patterns, and to explore what parameter values are more performant under which conditions.

### Aims and Objectives

This simulation study aimed to analyze the effect of different adherence estimation methods, sliding window parameters, and sample characteristics on the performance of a longitudinal clustering algorithm to (a) identify temporal adherence patterns from EHD and (b) classify individuals accurately into the identified clusters.

The objective of this study is to provide guidance for future longitudinal adherence studies using EHD.

## Materials and Methods

We conducted a simulation study to identify pre-defined groups with distinct longitudinal adherence patterns and to assess classification accuracy for different scenarios. To assess whether clustering on longitudinal trajectories offers advantages over simple clustering on group means, we compared performance of longitudinal classification to clustering with average Continuous Medication Availability (CMA) version 9 estimates for the whole observation period (Dima and Dediu, 2017). All simulations and cluster analyses were carried out on two systems: one cloud-based Microsoft Azure cluster of Virtual Machines running Linux and R version 3.5.1, and the other a dedicated dual Intel Xeon E5-2620 with 64GB RAM running Windows Server 2012 R2 and R 3.4.4 (R Core Team, 2018). Performance analyses were carried out with R version 3.5.0 running on Microsoft Windows 10 Pro x64. We followed published guidelines for the design and reporting of simulation studies in medical statistics (Burton et al., 2006).

### Data Generation

**FIGURE 1:**
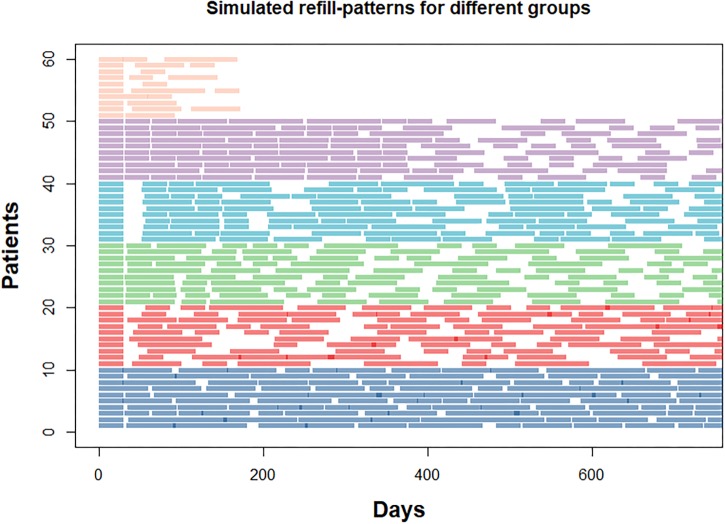
Refill-patterns for a sample of 10 individuals per group. Length of the bars represent supply duration in days. Groups are ordered from bottom (navy blue, group 1) to top (orange, group 6). Areas with higher saturation indicate refill overlaps.

We simulated refill histories for a single medication over an observation period of 720 days (2 years). This timeframe allows simulating realistic patterns observed for chronic treatments. To simulate successful treatment initiation, each individual had an initial fill for 30 days and at least one refill. Initiation happened on the same day for each individual. After the initial fill, refill durations of 30, 60, or 90 days were randomly sampled for each subsequent refill. Individuals were partitioned into one of six hypothetical groups with different longitudinal refill patterns (Figure 1):

• Group 1: *“High adherence”* with an average CMA9 of around 95%.
• Group 2: *“Erratic adherence”* with a median CMA9 between 50 and 90%.
• Group 3: *“Gradual decline”* with increasingly delayed refills.
• Group 4: *“Intermittent adherence”* with a change between high and low adherence at regular intervals.
• Group 5: *“Partial drop-off”* with high adherence initially and partial drop after some time.
• Group 6: *“Non-persistence”* with one or two refills after the initial fill and no refills afterward.

The parameters to generate the refill patterns for each group were tuned to achieve unique trajectories with distinct shapes over time similar to previously identified patterns (Franklin et al., 2013; Hargrove et al., 2017), e.g., groups 1, 3, 5, and 6. In addition, we added two other patterns to cover diverse rates of change (groups 2 and 4). Groups 1 (“*High adherence*”) and 6 (“*Non-persistence*”) were designed as control groups, as the individuals in these groups could be identified by calculation of average CMA alone; in our simulations, these groups represented 10% of the total sample size each. Groups 3–5 represented different temporal patterns but similar average CMA distributions so that identification of the correct group from average CMA was impossible. Group 2 served as “challenge” group with no underlying trend to use for clustering. With the exception of group 6, individuals persisted during the full observation period of 2 years. Details of the data generation can be found in the supplementary online materials on github^1^.

We generated a number of independent datasets to control for group size and refill durations, which might have an influence on the performance but are usually beyond the control of researchers and clinicians. The *group size* (proportion of individuals per group) can have an impact on the performance of the partitioning algorithm: if there are only few individuals in one group or the algorithm has difficulties to correctly identify individuals of a particular group, the performance may suffer. To control for this variation, group sizes were randomly sampled for each simulation with a minimum of 5% of the total sample size per group.

The *refill duration* (number of days covered by each refill) may vary based on medication type, health condition, healthcare system or other circumstances. To control for this variation, each refill duration was randomly sampled from a random sampling probability generated at the beginning of each simulation. For this simulation study, dispensing events covered 30-, 60-, or 90-day periods. These durations are consistent with the practice for long-term conditions in many healthcare settings. There was no minimum for each duration, meaning that a data set could consist of only 30-, 60-, or 90-day supplies (apart from the fixed initial 30-day supply).

### Cluster Analysis

To identify groups and classify individuals based on adherence trajectories, we used the R package “*kml*” (version 2.4.1), which provides an implementation of k-means designed to work specifically on longitudinal data (Genolini and Falissard, 2011; Genolini et al., 2015). In brief, the algorithm does not require prior information about groups, allows for the clustering of trajectories that do not follow polynomial or parametric functions, and avoids issues related to model selection. It features an implementation of the algorithm optimized for increased speed with default settings (Euclidean distance and 20 re-rolls with different starting conditions). In a direct comparison, *kml* showed equal or better performance compared to “Proc Traj,” a SAS implementation of “Group based trajectory modeling” frequently used to partition longitudinal data (Jones and Nagin, 2007) that has also been used in adherence research (Franklin et al., 2013, 2015; Lalic et al., 2018). To avoid overfitting to the dataset and to benefit from the fast implementation, we pre-specified the number of clusters to six (corresponding to the six pre-specified groups) and used *kml* with the default settings for all simulations. As a baseline comparison, we performed simple k-means clustering with the average CMA9 over the whole observation period with the default algorithm used by the *kmeans* function in R (Hartigan and Wong, 1979).

### Scenarios Investigated

#### Longitudinal Adherence Estimation

**FIGURE 2:**
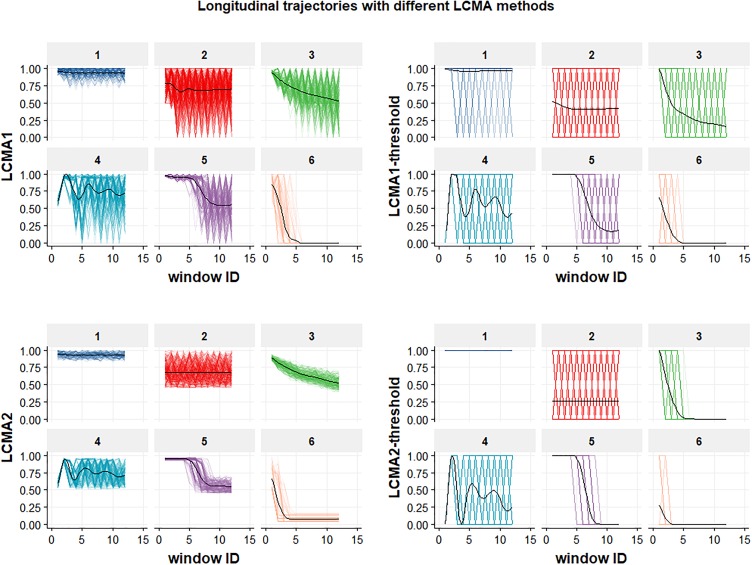
Longitudinal trajectories for the six groups in a sample of 1000 individuals. Each of the four panels shows the result of using one of the four CMA estimates (named on the vertical axis) to estimate adherence during the 24 non-overlapping 30-day-windows of the 2-year observation period (identified by window ID on the horizontal axis) for each of the six groups (identified by the numeric labels above each individual graph). Generalized additive models (GAMs) were used to fit mean trajectories (black lines).

Calculation of adherence from EHD allows only an estimation of medication availability over time, based on various assumptions (Arnet et al., 2016). For longitudinal adherence estimation, two methods based on different assumptions have been proposed for assigning an adherence estimate to each day, week or month of an observation period (Bijlsma et al., 2016; Franklin et al., 2013). The described methods mainly differ in their assumption about medication administration between refill events. For the purpose of this study, we defined the two different methods and their dichotomized versions as follows (Figure 2):

• Longitudinal Continuous Medication Availability (LCMA1) assumes that medication is administered as prescribed every day after a dispensing event until the supply is exhausted and not administered for the remaining days until a subsequent refill (Franklin et al., 2013). After each dispensing event, the consecutive number of days covered by the supply receive the value 1 and the remaining days until a subsequent refill receive the value 0. For each sliding window, the CMA estimate is the mean of the daily values within the window. This method does not allow for adherence values >100%, but oversupply may be carried forward.
• LCMA2 assumes that administration is evenly distributed over the time between refills (Bijlsma et al., 2016). Each day between refills receives the same adherence value, calculated as the duration of dispensed supply divided by the time until the next refill. This assumption is also implemented in CMA9 of AdhereR, an R package for the reproducible and transparent estimation and visualization of adherence from electronic healthcare data (Dima and Dediu, 2017). For each sliding window, the CMA estimate is the mean of the daily values within the window. This method allows for adherence values >100% if a refill occurs before an existing supply is exhausted, although oversupply may be carried forward (as in CMA9 of the AdhereR package).
• LCMA1-threshold and LCMA2-threshold dichotomize the output of LCMA1 and LCMA2, respectively, based on a threshold. For this simulation study, we used a threshold of 0.8, the most common threshold used in the literature (Karve et al., 2009). For each sliding window, the CMA estimate is 0 if the mean of the daily values within the window is below the threshold and 1 otherwise.

For this simulation study, we did not allow carryover, because individuals with a habit of early refills may be identified as a group with a distinct pattern. The way of handling oversupply should be decided based on the setting, health condition, and medication under investigation.

#### Sample Size

Adherence studies using EHD may involve between a few hundred and up to several hundred-thousand patients. With increasing number of patients, the computational costs to execute the partitioning algorithm increase, but it might affect performance as well. Due to computational limitations, we performed separate analyses with a limited number of window sizes and overlaps and compared performance between samples with 250, 500, 1000, 2500, 5000, 10,000, and 25,000 individuals to assess the impact of the sample size.

#### Window Size and Overlap

The sliding window size refers to the time covered by each window, e.g., 30 days. Subsequent windows “slide” forward with a defined lag time, creating overlaps of various degrees, e.g., 50% if windows of size 30 days slide forward with a lag time of 15 days. If window size and lag time are equal, windows do not overlap at all. The sliding window size and overlap might have an influence on the performance of the classification algorithm.

If the window size is small compared to the observation period, long-term trends may remain masked by noise and computation time may increase. With very long window sizes, shorter trends or gaps will get lost. The degree of overlap between windows mainly has an influence on the smoothness of the trajectory. With windows overlapping to a large degree, trajectories appear smoother. Larger overlaps also offer a possibility to regain some of the details when using longer window sizes, albeit at the cost of increased computational complexity.

In this simulation study, we assessed performance of the partitioning algorithm with various window sizes and overlaps. Window sizes covered 7, 14, and each multiple of 30 days up to the maximum duration of the observation period (720 days). For each window size, we assessed overlaps of 0–90% in 10% increments.

### Measures and Criteria to Evaluate Performance

For every scenario, we captured the original group assignments and classification results for individuals together with the parameters used (i.e., CMA estimate, sample size, window size, degree of overlap).

#### Identification of Groups

**FIGURE 3:**
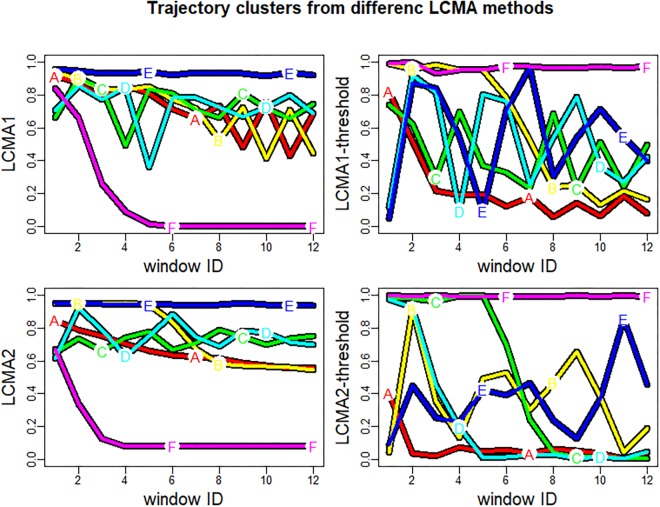
Trajectory clusters generated by *kml* for one sample of 1000 individuals. Each of the four panels shows the result of using *kml* with one of the four CMA estimates (named on the vertical axis) to estimate adherence during the 24 non-overlapping 30-day-windows of the 2-year observation period (identified by window ID on the horizontal axis). The colored lines (labeled **A–F**) represent the mean trajectories of the identified cluster. Note that the letters **A–F** do not necessarily correspond to groups 1–6.

Although the algorithm in our simulation study always partitioned individuals into six clusters with random labels from A–F, the predicted clusters did not necessarily resemble the pre-specified groups 1 to 6 (Figure 3). To compare performance between scenarios, we relabelled each cluster with the number of the best matching group. We defined the best matching group as the group with the highest representation in a given cluster. For example, the clusters A-F for LCMA2 in Figure 3 (bottom left panel) were relabelled to groups 3, 4, 5, 2, 1, and 6, respectively. If the predicted clusters did not correspond to the six pre-specified groups, the final number of predicted groups was smaller than six. For example, if the majority of the individuals in both of the predicted clusters A and B belonged to group 1, both clusters A and B received the label “1,” reducing the number of identified groups to 5.

#### Classification Accuracy

To assess classification accuracy, we computed the adjusted Rand Index (ARI), which is a commonly used measure for the similarity between two sets of clusters (Hubert and Arabie, 1985). The ARI takes a value between 0 (not better than random allocation) and 1 (perfect agreement with pre-specified group allocation). Because the number of correctly identified groups directly affects classification accuracy, we calculated two different ARIs: One restricted to the subset of the groups identified with the clustering algorithm (ARI) and another for the complete data set with all six groups (cARI).

For each CMA estimate, we assessed the impact of sample size, window size, and overlap on the identification of pre-specified groups and on classification accuracy and calculated overall performance differences between CMA estimates using pairwise ANOVA and Tukey’s range test. To understand performance differences for each group, we created confusion matrices for each CMA estimate to visualize the classification accuracy.

## Results

We simulated 50 data sets per sample size (350 in total) to assess impact of sample size on performance. Sample size did not have a substantial impact on overall classification accuracy (Spearman’s Rho = 0.04, *p*-value = 0.5), and increased computational costs considerably. For the final analysis, we simulated 100 data sets with 1000 individuals and investigated 1040 different scenarios (four adherence estimates, 26 window sizes, and 10 degrees of overlap per window size) for each data set. Mean refill duration was 60.58 days (IQR: 53.83–67.62) with a mean interval of 92.4 days (IQR: 84.10–103.07) between refill events.

### Performance Analysis

Cluster analysis with LCMA2 outperformed every other method irrespective of sliding window parameters in overall performance, correct identification of groups, and classification accuracy. Pairwise comparison of overall cARI showed a relative advantage of 0.12–0.22 for LCMA2 compared with other estimates. All differences were highly significant using pairwise ANOVA and Tukey’s range test except for the dichotomized estimates (Table 1).

**Table 1:** Pairwise comparison of overall cARI between CMA estimates (100 simulations of 1000 individuals).

CMA comparison	Diff (95% CI)	*p* adj
LCMA2 : LCMA1-thr	0.22 (0.18–0.25)	< 2.2 × 10^-16^
LCMA2 : LCMA2-thr	0.20 (0.17–0.24)	< 2.2 × 10^-16^
LCMA2 : LCMA1	0.12 (0.08–0.15)	< 2.2 × 10^-16^
LCMA1 : LCMA1-thr	0.10 (0.07–0.14)	< 2.2 × 10^-16^
LCMA1 : LCMA2-thr	0.09 (0.05–0.12)	< 1.2 × 10^-8^
LCMA2-thr : LCMA1-thr	0.02 (–0.02–0.05)	0.65

*Diff: Difference in overall adjusted Rand Index, CI: confidence interval, *p* adj: P-values adjusted for multiple comparison, thr: Threshold, indicating the dichotomized LCMA estimates.*

#### Impact of Window Size and Overlap on Overall Performance

**FIGURE 4:**
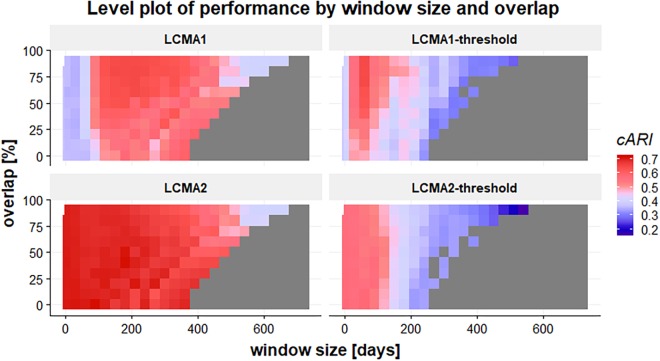
Level plot of the cARI in relation to window size (horizontal axis, in days) and overlap (vertical axis, in percent) for 100 simulations of 1000 individuals. The four panels of the graph represent the different LCMA estimates (identified by the panel title). The gray area in the lower right corner shows where the longitudinal classification algorithm failed because there were less than two windows or less than six distinct trajectories to form clusters. Colors range from blue (low cARI) to red (high cARI).

**FIGURE 5:**
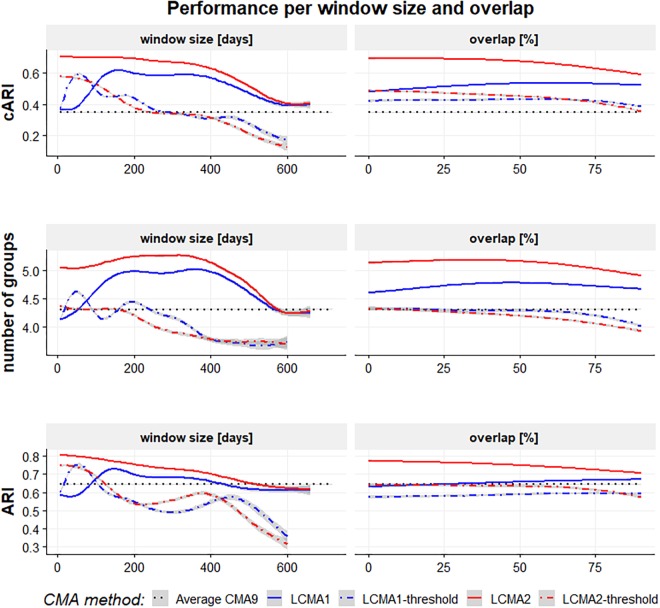
Performance with different CMA methods per window size (in days, left-hand side panels) and overlap (in percent, right-hand side panels) for 100 simulations of 1000 individuals. The vertical axes are adjusted Rand Index for all groups (top panels), average number of correctly identified groups (mid panels) and adjusted Rand Index for the identified groups (bottom panels). The black dotted line represents the performance of simple *k*-means clustering on the average CMA9 over the whole observation period.

Window size and overlap affected overall performance with all CMA estimates, albeit in different ways (Figure 4 and top row of Figure 5). LCMA2 consistently showed the best overall performance, with the highest cARI values for smaller window sizes and overlaps. LCMA1 showed the worst performance for smaller window sizes up to 100 days and reached peak cARI values with window sizes of 150–200 days and large overlaps. Performance of both dichotomized estimates was generally better with short window sizes and decreased rapidly with larger window sizes. Although performance of LCMA2 also decreased with larger window sizes, it remained relatively stable up to around 360 days (half of observation period). Classification with the dichotomized estimates required at least three windows, which was due to the requirement to generate six different clusters and the limited options to form six distinct trajectories with dichotomized values. Consequently, the possible window sizes were restricted, e.g., to 240 days for non-overlapping windows compared to 360 days for the continuous LCMA-methods).

With LCMA1 and LCMA2, the number of correctly identified groups was higher for larger window sizes between 180 and 360 days and overlaps between 25 and 70% (Figure 5, middle row). However, the effect was more pronounced with LCMA1. Overall, with both dichotomized versions (LCMA1-threshold and LCMA2-threshold) the number of correctly identified groups was lower than for clustering on average CMA9. With LCMA2 and window sizes below 420 days, *kml* correctly identified at least five of the six groups in over 90% of the scenarios.

With LCMA2, classification accuracy for the correctly identified groups (ARI) was best with short window sizes and small overlaps (Figure 5, bottom row). For the other LCMA methods, the curve for classification accuracy was similar to that for correctly identified groups.

#### Classification Accuracy per Group

**FIGURE 6:**
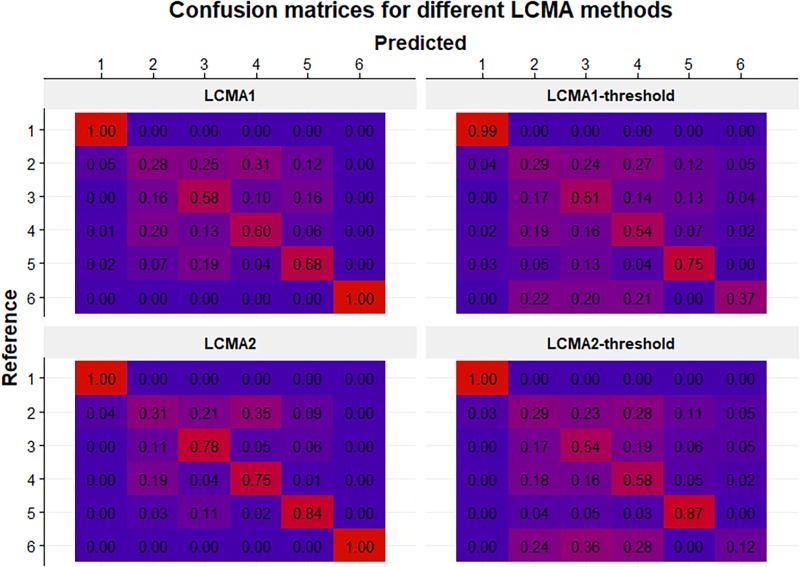
Confusion matrices for each CMA estimate (identified by the panel titles) for 100 simulations of 1000 individuals. Rows represent the pre-specified (i.e., reference) groups (from 1 to 6 in top to bottom order) and columns represent the predicted clusters after relabelling (from 1 to 6 in left to right order). Numbers in cells indicate the mean frequency with which patients from the reference group were classified in the predicted cluster (i.e., 0.25 in the 2:3 cell in the top panel means that on average 25% of the patients in the reference group 2 were classified into cluster 3). Colors range from blue (0%) to red (100%).

Classification accuracy varied not only between CMA estimates and sliding windows parameters, but also between the six pre-specified groups (Figure 6). The reference group 1 (*consistent adherence*) was correctly identified with all methods, including CMA9. Reference group 6 (*non-persistence*) was identified with LCMA1 and LCMA2 (and CMA9), but not with the dichotomized variants. For this group, LCMA2-threshold showed the weakest accuracy with less than 20% of non-persistence correctly identified. As expected, group 2 (*erratic adherence*) was the most problematic with an accuracy of around 30% for all estimates. For groups 3–5, LCMA2 reached an overall classification accuracy of around 80%, unrivaled by any other estimate. In comparison, clustering with CMA9 reached an accuracy of around 50% for these groups.

**FIGURE 7:**
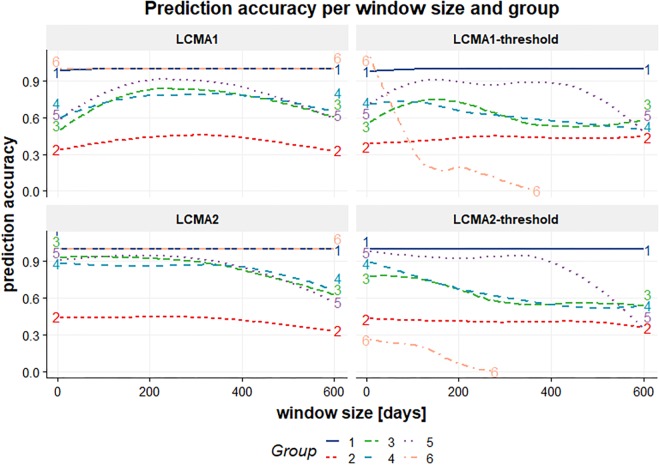
Classification accuracy (vertical axis) per window size (horizontal axis, in days) and group (colored lines) for each CMA estimate (individual panels identified by their title) for 100 simulations of 1000 individuals.

While classification accuracy was consistent between groups over window sizes for LCMA1 and LCMA2, accuracy for different window sizes varied between groups with the dichotomized estimates (Figure 7).

### Choice of Optimal Parameters

In our simulation study, optimal sliding window parameters varied between CMA estimates (Table 2). The continuous estimates LCMA1 and LCMA2 reached optimal performance at larger window sizes of 150 and 90 days, respectively. The dichotomized versions required shorter windows of 60 days. Both LCMA2 estimates performed better with non-overlapping windows. In contrast, LCMA1 estimates required larger overlaps of 80% for optimal performance. With LCMA2 and non-overlapping windows of 90 days, *kml* correctly identified group membership for an average 84.4% of individuals in 100 independent simulations.

**Table 2:** Optimal parameters and performance per CMA method, based on 100 simulations of 1000 individuals.

	CMA9	LCMA1	LCMA1-thr	LCMA2	LCMA2-thr
Window size	–	150	60	90	60
Overlap	–	80%	70%	0%	0%
Mean cARI [95% CI]	0.35 [0.33–0.36]	0.65 [0.62–0.68]	0.65 [0.62–0.68]	0.72 [0.69–0.74]	0.58 [0.55–0.62]
**Classification accuracy [95% CI]**					
Consistent adherence	100%	100%	100%	100%	100%
	[100–100%]	[100–100%]	[100–100%]	[100–100%]	[100–100%]
Erratic adherence	18%	29%	27%	33%	29%
	[13–23%]	[23–35%]	[21–34%]	[28–39%]	[23–35%]
Gradual decline	53%	74%	65%	85%	77%
	[45–62%]	[66–82%]	[57–74%]	[77–91%]	[70–85%]
Intermittent adherence	51%	70%	75%	76%	74%
	[43–60%]	[62–77%]	[68–83%]	[69–83%]	[67–81%]
Partial drop-offs	51%	81%	84%	86%	89%
	[43–59%]	[75–88%]	[77–90%]	[80–92%]	[85–95%]
Non-persistence	100%	100%	89%	100%	21%
	[100–100%]	[100–100%]	[84–95%]	[100–100%]	[14–28%]
Overall	66%	80%	79%	84%	75%
	[65–68%]	[79–82%]	[78–81%]	[83–86%]	[73–76%]

*A classification accuracy of 100% indicates that the clustering algorithm correctly identified all individuals of a pre-allocated group. cARI: adjusted Rand Index for all groups, CI: confidence interval.*

## Discussion

### Overview

Classifying patients based on their long-term medication use behaviors could prove useful in numerous clinical settings, to understand reasons for low adherence to prescribed treatments and decide on treatment and behavioral support needs. In addition, researchers may use trajectory-based models to classify subjects based on properties emerging from empirical data instead of *a priori* criteria such as thresholds. Such classification needs to rely on methods with proven performance regarding the identification of underlying patterns and the classification accuracy. To our knowledge, this is the first simulation study to systematically analyze the effect of different adherence estimates, sample size, and sliding window parameters on the performance of a longitudinal classification algorithm. Our study showed that compared to other methods, LCMA2 is the most appropriate method for calculating medication availability trajectories to use for longitudinal clustering. With LCMA2, a longitudinal *k*-means algorithm reliably identified six distinctive adherence patterns from electronic healthcare data during an observation period of 2 years. In addition, it generates visualizations that represent most accurately the individual adherence trajectories. We recommend using LCMA2 in longitudinal adherence studies to identify and explore different time-varying adherence patterns and to visualize individual adherence trajectories to assist with clinical decisions.

In contrast to LCMA1 and most other CMA methods, LCMA2 does not assume 100% use until the supply is exhausted. In the case of longitudinal adherence analysis, this assumption has been questioned before (Bijlsma et al., 2016). While it may have only a minor impact on average adherence estimates over long periods, it strongly affects estimates for periods shorter than the interval between two refills, as it is the case with sliding window analyses. Our simulation study shows that performance with LCMA1 is poor for window sizes below 90 days, which coincides with the average interval between dispensing events in our simulated data sets. However, performance remained lower compared with LCMA2 even for larger window sizes, although larger overlaps improved performance noticeably. In contrast, window sizes up to one year (half of the observation period) and overlap had only a minor impact on performance with LCMA2.

Performance with the dichotomized versions of both LCMA was lower than with the continuous estimates, which illustrates the loss of information consequent to this decision. The dichotomization on a threshold makes it difficult to distinguish non-persistence from implementation below the threshold. This distinction between implementation and non-persistence is crucial to understand reasons for low adherence and for decision-making regarding medical treatment or behavioral support. Thus, we recommend against the use of thresholds in trajectory-based models. However, dichotomizing adherence estimates of particular subgroups identified in cluster analysis may be appropriate, e.g., for erratic adherence (group 2 in our study) or to identify the exact moment when individuals with delayed adherence (group 3), intermittent adherence (group 4) or partial drop-off (group 5) cross a certain threshold.

Sample size did not have a substantial effect on overall performance. Hence, we performed our study with 1000 individuals per simulated dataset. However, real data sets may consist of much smaller or larger samples. With smaller samples, the number of individuals per group might be too small to appear as a separate cluster. Classification with more individuals requires more computational resources. The requirements for computational resources increase not only with sample size, but also with the length of the trajectories (e.g., number of sliding windows) included in the analysis. For a trajectory length of 101 windows, clustering with *kml* was possible for a maximum of 40,000 individuals on a typical consumer machine with 8 GB of RAM and required over 20 h to complete (Genolini et al., 2015). Thus, clustering on larger data sets benefits from a larger window size and short overlaps. With the optimal parameters for LCMA2 in our study (non-overlapping windows of 90 days), an observation period of 2 years results in trajectories of length 8, which should not take more than 7 h to classify with *kml* on a typical machine for sample sizes of up to 40,000.

### Advantages

Our methods for estimating longitudinal adherence based on EHD and the performance analysis in a simulation study have several advantages.

First, we calculated LCMA2 for intervals between two refills instead of the three dispensing events proposed by Bijlsma et al. (2016). This ensures the calculation of an adherence estimate even if there are less than three dispensing events (e.g., some individuals in group 6 of our simulation study). While stabilizing the adherence estimate and reducing variance, the Bijlsma et al. approach may mask temporal variation, e.g., if the interval between the first and second dispensing event is a lot longer or shorter than between the second and third. With larger window sizes, we achieved a similar form of stabilizing adherence estimates over multiple dispensing events. Nevertheless, with our method of calculating LCMA2 between two dispensing events, time-varying adherence patterns were reliably identified with short window sizes covering not more than the interval between two dispensing events. On the other hand, short-term temporal variation can also mask true underlying adherence patterns, e.g., with early refills resulting in overlapping supplies. If this is a concern, we recommend to carry-over oversupplies before adherence estimation.

Second, we separately assessed performance regarding the identification of groups and classification accuracy. With this approach, we were able to separate the issue of cluster selection (identification of cluster centers) from the classification accuracy (identification of cluster boundaries).

Third, we simulated a large number of independent data sets and compared classification performance over a wide range of sliding windows parameters. This kind of analysis would not be possible with real datasets, because cluster analysis is by definition always exploratory. To identify optimal parameters for the analysis of a specific data set, we recommend performing a simulation study similar to the one outlined here, but adapted to the data set under investigation. Researchers may simulate adherence patterns, group sizes, and refill durations based on population characteristics and study objectives to identify optimal parameters. To facilitate such studies, researchers may refer to the source code released under a GNU General Public License v3 on github^2^.

### Limitations

We report several limitations relating to cluster analysis in general and to our simulation study in particular.

First, *kml* (and most other trajectory-based models) group trajectories together that are similar at given time points. As a result, the shape of the trajectory might be less important than the time at which change occurs. For this simulation study, the change in adherence happened around similar times for all individuals in a particular group. To arrive at meaningful results in real-world scenarios, trajectories should align with specific events of interest, e.g., the start of a treatment, hospitalization, or another clinical outcome. In some instances, however, the shape of the adherence trajectory might be more important than the time at which it changes, e.g., when the time of initiation of a treatment is not known. For these instances, other classification methods such as a shape-respecting version of *kml* (*kmlShape*) might offer advantages (Genolini et al., 2016).

Second, *kml* is non-parametric, which might be an advantage in some situations, but can be a limitation in others. Due to the lack of an underlying model, it is not possible to test a fit or specify a likelihood for group membership of individuals. (Semi-)parametric methods such as group-based trajectory modeling (Nagin and Odgers, 2010) and other variants of finite mixture models have these properties, which can be useful especially if clusters are not well separated. Nevertheless, our results concerning the choice of LCMA methods and sliding windows parameters should in principle be valid for these methods as well.

Third, we did not address the issue of cluster selection, although the number of correctly identified groups had a major impact on performance. Unlike in our simulation study, the “true” number of groups in a data set is usually unknown. Identifying the correct number of clusters is a long-standing issue in cluster analysis (Everitt et al., 2001). One possibility is to perform cluster analysis with varying number of groups and then select the “best” number of clusters based on a quality criterion (Milligan and Cooper, 1985). With LCMA2, the number of correctly identified groups was highest for window sizes between 200 and 360 days, but classification accuracy was highest for short window sizes and linearly decreased with longer window sizes. Hence, overall performance would be highest if the “true” groups could be identified reliably with short window sizes. This could be achieved by tuning the starting conditions for the clustering algorithm or performing classification with pre-set cluster centere based on clusters previously identified with longer window sizes.

Fourth, we did not systematically analyze the impact of refill duration and group size on classification performance. It appears that the window size for optimal performance with LCMA2 coincides with the mean interval between refills, but this should not serve as a recommendation without further verification. Nevertheless, overall performance with LCMA2 was robust over a wide range of parameters.

Finally, the results of our simulation study are difficult to validate with real data, because cluster analysis is always exploratory. We strived to simulate realistic refill patterns for medications intended for chronic use. At the same time, we needed patterns that (a) were distinct enough to allow partitioning into groups and (b) had similar average adherence (otherwise, one could just group by average adherence, which would not be useful to answer the study question). Hence, the parameters were a trade-off between internal and external validity. However, the simulated refill patterns and resulting adherence trajectories have been observed previously in real data sets (Franklin et al., 2013, 2016; Hargrove et al., 2017). Because of the clear and highly significant advantage of LCMA2 over a wide range of sliding windows parameters, we are confident that our results can be generalized to many other real-world settings.

### Outlook

To correlate temporal adherence patterns with other outcomes, such as time of hospitalization, exacerbations or illness progression, or discontinuation with treatment, joint modeling techniques could combine adherence trajectories from a sliding-windows approach with a relative risk model (Sweeting and Thompson, 2011). *Kml* offers this functionality with its *kml3d* package (Genolini et al., 2015). This approach could be applied to other adherence estimates derived from electronic monitoring or self-report (especially joint analysis).

To facilitate longitudinal adherence analysis from EHD, the AdhereR package implements sliding-windows capabilities using different (even user-defined) adherence estimates (Dima and Dediu, 2017). The LCMA2 used in this study is the equivalent to sliding windows using CMA9 in AdhereR, albeit the standard CMA9 accounts for carry-over. As AdhereR is under active development, we will add additional options for longitudinal adherence research in the future based on our own research and user feedback.

## Conclusion

The choice of CMA estimates and sliding window parameters has a major impact on the performance of a clustering algorithm to identify distinct longitudinal adherence trajectories. We recommend (a) assuming constant adherence between refills, (b) avoiding dichotomization based on a threshold, and (c) exploring optimal sliding windows parameters in simulation studies or selecting shorter non-overlapping windows for the accurate and robust identification of different adherence patterns from electronic healthcare data.

## Author Contributions

SA and DD programmed the simulations. SA performed the analyses and wrote the first draft of the manuscript. AD and DD wrote sections of the manuscript. All authors contributed conception and design of the study, manuscript revision, read and approved the submitted version.

## Conflict of Interest Statement

The authors declare that the research was conducted in the absence of any commercial or financial relationships that could be construed as a potential conflict of interest. The handling Editor declared a shared affiliation, though no other collaboration, with one of the authors SA at the time of review.

## Footnotes

1 github.com/Masswear/BeyondThresholds/

2 github.com/Masswear/BeyondThresholds/
